# Short-Range Charge Transfer in DNA Base Triplets: Real-Time Tracking of Coherent Fluctuation Electron Transfer

**DOI:** 10.3390/molecules28196802

**Published:** 2023-09-25

**Authors:** Lixia Zhu, Qi Li, Yongfeng Wan, Meilin Guo, Lu Yan, Hang Yin, Ying Shi

**Affiliations:** Institute of Atomic and Molecular Physics, Jilin University, Changchun 130012, China; lixia21@mails.jlu.edu.cn (L.Z.); lqi22@mails.jlu.edu.cn (Q.L.); wanyf20@mails.jlu.edu.cn (Y.W.); guoml21@mails.jlu.edu.cn (M.G.); yan_lu21@mails.jlu.edu.cn (L.Y.); yinhang@jlu.edu.cn (H.Y.)

**Keywords:** nuclear-electron vibronic coupling, coherent electron transfer, Ehrenfest dynamics, periodic oscillation, DNA base triplet

## Abstract

The short-range charge transfer of DNA base triplets has wide application prospects in bioelectronic devices for identifying DNA bases and clinical diagnostics, and the key to its development is to understand the mechanisms of short-range electron dynamics. However, tracing how electrons are transferred during the short-range charge transfer of DNA base triplets remains a great challenge. Here, by means of ab initio molecular dynamics and Ehrenfest dynamics, the nuclear–electron interaction in the thymine-adenine-thymine (TAT) charge transfer process is successfully simulated. The results show that the electron transfer of TAT has an oscillating phenomenon with a period of 10 fs. The charge density difference proves that the charge transfer proportion is as high as 59.817% at 50 fs. The peak position of the hydrogen bond fluctuates regularly between −0.040 and −0.056. The time-dependent Marcus–Levich–Jortner theory proves that the vibrational coupling between nucleus and electron induces coherent electron transfer in TAT. This work provides a real-time demonstration of the short-range coherent electron transfer of DNA base triplets and establishes a theoretical basis for the design and development of novel biological probe molecules.

## 1. Introduction

The photoinduced short-range electron transfer of DNA base triplets plays an important role in clinical diagnosis, in the identification of biological bases, and in gene replication and mutation [1,2,3,4,5]. There is increasing research focused on exploring the mechanism of short-range electron transfer [6,7,8]. The short-range coherent superexchange charge transfer proposed by Giese et al. can perfectly explain the continuous electron transfer process of DNA base triplets [9]. Subsequently, it has been reported that the sustained electron transfer in DNA base triplets consists of a series of short-range tunneling processes [10]. It follows that tracking the electron moving of the DNA base triplets is absolutely helpful for understanding the short-range electron transfer.

Thymine-adenine-thymine (TAT) is a representative base triplet, which consists of one charge donor and two charge acceptors [11,12,13,14,15,16]. Adenine (A) is oxidized and can be used as a hole carrier, while thymine (T) is reduced and regarded as an electron carrier [17,18]. T and A form a dimer through the Watson–Crick structure, and the third base T is parallel to A and connected by hydrogen bonds to form a stable base triplet structure [19,20,21,22]. At present, the charge transfer of TAT base triplets at short-range is thought to be caused by superexchange [23,24]. When superexchange predominates over charge transfer, the hole directly tunnels from the donor to the acceptor using the base pairs as virtual states [25]. Renaud et al. simulated charge transfer with less than seven AT base pairs, demonstrating that hole migration does, indeed, occur through a superexchange mechanism [26]. Until now, the subject of TAT short-range charge transfer research has concentrated on the hole migration. It is worth noting that short-range electron transfer also plays an irreplaceable role in gene replication and mutation. When a DNA strand contains fewer than four or five bases, the charge transfer process can be considered short-range [27]. However, few reports have been made about how electrons are transferred in the short-range charge transfer of a TAT base triplet, which prevents the further exploration of the basic mechanism of genetic information. It is not conducive to the application of the biological electronic devices that recognize DNA bases in clinical diagnosis.

Here, the nuclear–electron interaction during short-range electron transfer at TAT is simulated using ab initio molecular dynamics in conjunction with Ehrenfest dynamics. It is discovered that the electron transfer of TAT exhibits a distinct periodic vibration behavior with a duration of 10 fs. The time-dependent Kohn–Sham eigenvalues, N-H bond vibration, and hydrogen bond strength are calculated with time. The evolution processes of charge density differences, transition density matrix, and hole–electron distribution at critical time points are presented. In addition to classifying the localized excitation and charge transfer excitation, the hole–electron separation degree and overlap degree are quantified. Moreover, the calculated real-time Marcus–Levich–Jortner theory gives the free energy and reorganization energy at different times. The results demonstrate that the strong vibrational nucleus–electron coupling causes the coherent migration of the electron in the TAT base triplet. The findings add to the investigation of the coherent effects in related biological systems and offer a beneficial comprehension of the coherent electron transfer dynamics of DNA base triplets.

The current work mainly focuses on exploring the long-range hole transfer process of DNA base triplets, but the electron transfer process under short-range conditions is rarely reported. The aim of this study is to explore the physical mechanism of short-range charge transfer in TAT base triplets. It is revealed that the nuclear–electron vibronic coupling induces the coherence of the short-range charge transfer of TAT base triplets.

The paper is organized as follows. In the next section, we show the trend of molecular orbital energy over time and quantify the distribution of electrons and holes. The Marcus–Levich–Jortner theory is applied to reveal the mechanism of TAT short-range electron transfer. The third section summarizes the content of the paper, and the last section describes the calculation method in detail.

## 2. Results and Discussion

### 2.1. Photoinduced Charge Transfer

**Figure 1:**
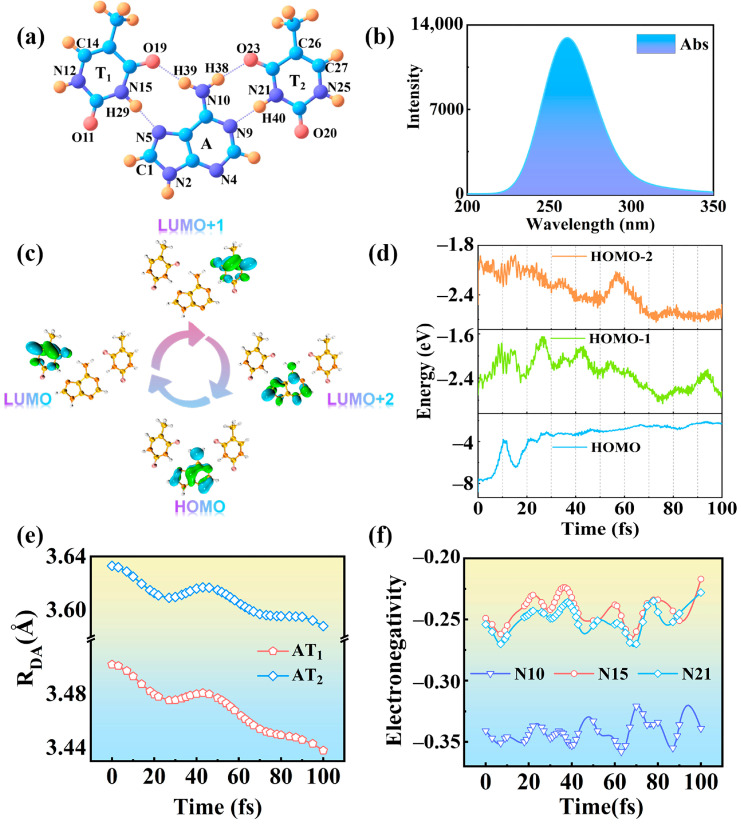
(**a**) Structure of TAT base triplet. Blue: C; Orange: H; Pink: O; Purple: N. (**b**) Absorption spectrum of TAT base triplet. (**c**) The molecular orbitals involved in charge transfer. Blue for holes, and green for electrons. (**d**) Time evolution of molecular orbitals LUMO, LUMO + 1, and LUMO + 2 involved in coherent charge transfer process. (**e**) The distance between the donor and acceptor varies with time. (**f**) The electronegativity of N_10_, N_15_, and N_21_ over time.

The structure of TAT is displayed in Figure 1a. To more faithfully simulate the electron transfer dynamics of TAT, we fixed N_4_, N_12_, and N_25_. Such fixing can prevent complicated translational damage to the equilibrium position as well as maintain the relative distance between the donor and acceptor. Similar research has demonstrated that, if fixed methods are not employed, the electron transfer process will be impeded [28]. Various functionals and basis sets are selected to ensure the accuracy of the simulation. Table S3 shows that the TAT absorption peak calculated by B3LYP/TZVP level is in good agreement with the experimental results (260 nm) [29,30]. The absorption spectrum of the TAT base triplet is shown in Figure 1b. It is proved that the molecular structure is simulated, and that the functional and basis set selected are reasonable and effective. The frontier molecular orbitals provide a very clear view of the distribution of charge in different molecular orbitals [31,32]. Figure 1c reveals the molecular orbitals involved in the photoinduced charge transfer process of TAT. Upon excitation, the electron on donor (A) begins to change the occupied orbitals. The HOMO distribution decreases, the LUMO distribution increases, the LUMO + 1 distribution increases, and, eventually, the charge relocates in the LUMO + 2 orbital. The HOMO at A drops to 0 and the LUMO, LUMO + 1 at T_1_ and T_2_ increase to 1, indicating that TAT is locally excited. When the Franck–Condon region is in transient excitation, the system begins to move towards a more desirable geometry. So the change in molecular orbitals from LUMO, LUMO + 1 and LUMO + 2 may be related to electron transfer.

The evolution of orbital energy over time can clearly clarify the whole process of coherent electron transfer [33,34,35,36,37]. Figure 1d is a schematic diagram of the time evolution of the orbital energy of TAT. At the beginning of electron transfer, the part far away from Franck-Condon region will undergo a rapid relaxation process. So that the orbital energies of LUMO + 1 and LUMO are closer to each other, which corresponds to the occurrence of nuclear motion. As the orbital energy approaches, the orbital energies oscillate regularly over time, with a period of about 10 fs. The vibrational nuclear–electron coupling makes the orbital energy change periodically, which leads to the occurrence of coherent electron transfer in TAT. The electron coupling strength ($H_{DA}$) changes in response to nuclear motion. The $H_{DA}$ and the distance between the donor and the acceptor ($R_{DA}$) are closely related. The specific relationship is as follows: $H_{DA}$ and $R_{DA}$ form an e-exponential relationship, which can be obtained using the Marcus theoretical formula [38]:

(1) $$H_{DA}\propto \exp(-\beta R_{DA})$$

where β marks the exponential decay constant. The change in $R_{DA}$ over time is displayed in Figure 1e. Obviously, the decreasing distance of $R_{DA}$ proves that the nuclear–electron coupling strength increases regularly with time. It suggests that there is a strong coupling between the nucleus and the electron of the TAT base triplet. In addition, it is worth noting that the distance of AT_1_ is significantly smaller than that of AT_2_, which is caused by the asymmetry of the TAT structure. In order to determine which type of bond vibration coupling promotes coherent electron transfer, infrared vibrational spectra are plotted, as shown in Figure S2. It is found that the bonds located around 3000 cm^−1^ move periodically with time, and these bonds belong to the N-H bonds near the hydrogen bond grid.

Figure 1f plots the electronegativity variation trend of atoms near the hydrogen bond grid. At 0 fs, the electronegativity of N_10_ is greater than that of N_15_ and N_21_, indicating that the donor (A) has the strongest ability to attract electrons and the charge is completely distributed on the donor (A). At 9 fs, the electronegativity of N_15_ and N_21_ increases, stating that the acceptor (T_1_, T_2_) has a stronger ability to attract electrons, and there is an obvious charge distribution on the acceptor. The electronegativity of 40 fs is similar to that of 9 fs, but the electronegativity of N_21_ is slightly larger than that of N_15_, manifesting that the charge distribution on T_2_ is larger than that on T_1_. At 50 fs, the electronegativity of N_10_ is smaller, while the electronegativity of N_15_ and N_21_ is basically equal, and both show a trend of increasing. This means that the distribution of charge on the donor (A) decreases, and the distribution of charge on the acceptor (T_1_, T_2_) is increased and uniform. In short, in the period from 0 fs to 100 fs, the electronegativity values of N_10_, N_15_, and N_21_ atoms all show regular oscillations with a period of about 10 fs.

### 2.2. Reduced Density Gradient

**Figure 2:**
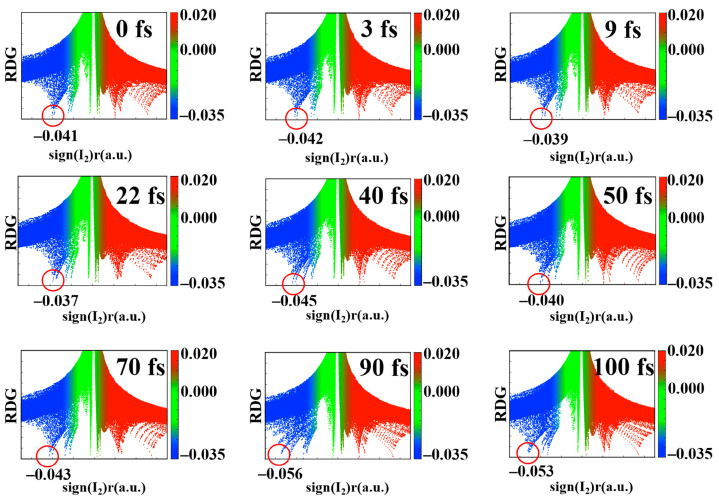
Graph of the function values of the TAT base triplet. The assignment of each peak on the gradient isosurface. The red circle marks the peak of the hydrogen bond.

Regular changes in electronegativity will inevitably lead to change in the strength of the hydrogen bond. The evolution of the reduced density gradient (RDG) of electron density with time is an intuitive way of showing the change in hydrogen bond strength [39,40]. The hydrogen bond strengths of the TAT base triplet at different times are depicted in Figure 2 and the oscillating process of the N-H bonds near the hydrogen bond grid are observed in Figure S3. Blue represents the weak hydrogen bond interaction. Red denotes steric hindrance. The values around green represent van der Waals forces. It is observed that the hydrogen bond peak is at −0.041 at 0 fs. From 3 fs to 9 fs, the peak position of the hydrogen bond shifts from −0.042 to −0.039, and the corresponding hydrogen bond is stretched. From 22 fs to 40 fs, the system has a peak position from −0.037 to −0.045. The strengthening of the hydrogen bond corresponds to the compression of bonds such as N_21_-H_40_ and N_10_-H_39_. The hydrogen bond peak of 50 fs is again shifted to the right to −0.040. This denotes that the hydrogen bond strength decreases and the corresponding N_15_-H_29_ and N_10_-H_38_ are in a tensile state. From 70 fs to 90 fs, the hydrogen bond strength increases again, and the position of the peak corresponds to a left shift from −0.043 to −0.056. The peak position stabilized at about −0.05 at 100 fs. During 0–100 fs, the peak position of the hydrogen bond fluctuates regularly between −0.040 and −0.056. The periodic changes in hydrogen bond strength and bond length once again confirm that the charge transfer of TAT base triplets is coherent.

### 2.3. Charge Density Difference Analysis

**Figure 3:**
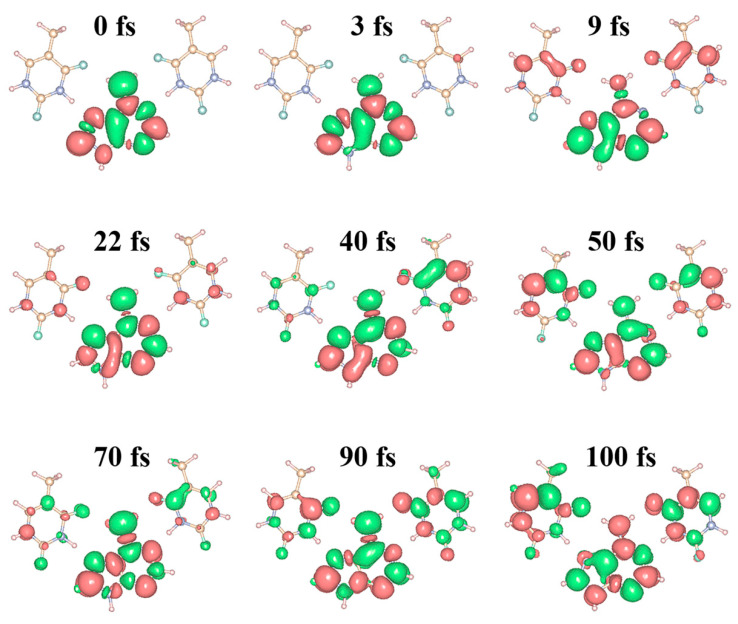
Visualizing the time-dependent evolution of coherent charge transfer in TAT base triplets. Simulation of CDD evolution over time. The pink represents electrons, and the green represents holes.

To quantify the dynamic coherent charge transfer, we visualize the charge density difference (CDD) using a three-dimensional real space analysis method. Figure 3 and Movie S1 describe the time-dependent distribution of the coherent charge of TAT, and the specific proportion of charge transfer is shown in Table S4. The decreasing regions of CDD belong to photoinduced holes and are shown in green. The regions of increased CDD correspond to coherent electron transfer and are represented in red. The charge is completely concentrated on donor A at 0 fs. The electron is gradually transferred to the acceptor T_1_ at 3 fs, marking the start of coherent electron transfer. From 3 to 9 fs, more charge is being transferred from A to T_1_ and T_2_. It is worth noting that the charge distributions at T_1_ and T_2_ are very similar and uniform, indicating that the coherent electron transfer of TAT is synergistic rather than competitive. At this point, the charge transfer proportion increases to 25.102%. The charge is almost all concentrated on A at 22 fs, which proves that a new cycle has entered at this time. At 40 fs, the charge is gradually transferring from the donor to the acceptor, but the charge distribution at T_1_ is more than that at T_2_. This expresses that the distribution of the charge at the acceptor is not simultaneous. At 50 fs, more charge is found at T_1_ and T_2_, which attests to the fact that coherent charge transfer is already near the end of a cycle. The corresponding charge transfer proportion is the largest at 59.817%. At 70 fs, the charge of A increases and the charge of T_1_ and T_2_ decrease again. However, at 90 fs, most of the charge is transferred to T_1_ and T_2_ again, and the charge transfer proportion is at 27.363%. At 100 fs, the charge coexists at the donor and acceptor, and the charge distribution is widest at the acceptor. Over time, the charge delocalizes between the donor and acceptor. The oscillation time of Figure 1d,f is compatible with the periodic change in CDD. Furthermore, we notice that the charge is distributed around the heavy atoms during the entire periodic oscillation from 0 fs to 100 fs (Supplementary Materials). Therefore, a better grasp of the microscopic mechanism of coherent charge transfer can be gained by visualizing the charge distribution around heavy atoms.

### 2.4. Transition Density Matrix Analysis

**Figure 4:**
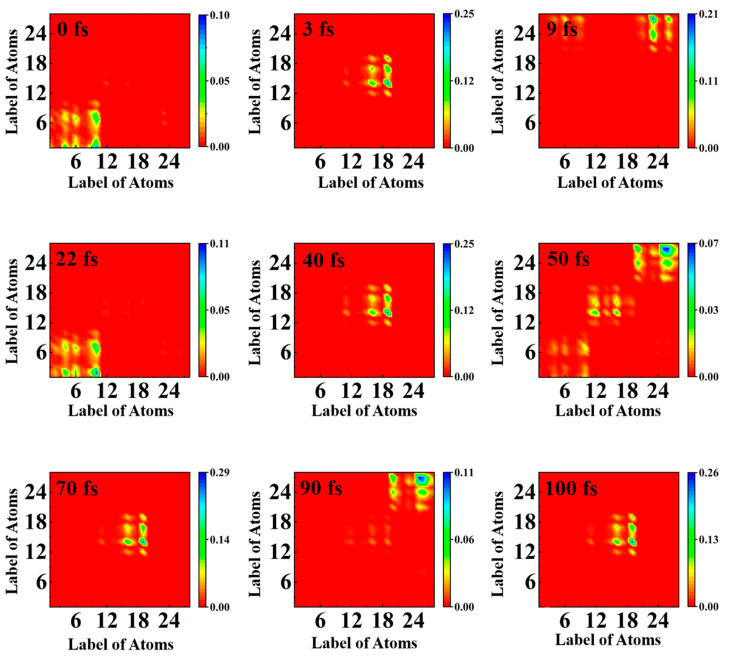
The distribution of holes and electrons with time is observed by using the two-dimensional real space method. The TDM heat maps of the electron’s movement with time.

The electron–hole coherence can be accurately observed by analyzing the transition density matrix (TDM) in two-dimensional real space [41,42,43,44]. Since hydrogen does not contribute much to the overall charge transfer process, the effect of hydrogen is ignored. Figure 4 depicts the change in TDM in TAT. The abscissa is the source of electron (green). The ordinate represents the potential location of the electron during the transition (blue). At 0 fs, element (10, 1) accounts for the largest proportion, which expresses the electron transition from N_10_ to C_1_, the charge is completely populated on donor (A), and the electron–hole coherence is stronger at A. At 3 fs, the element (19, 14) accounts for the largest proportion, representing the transfer of electrons from O_19_ to C_14_, and coherent charge transfer begins to occur. This result is consistent with Figure 3. At 9 fs, the proportion of (23, 27) is the largest, showing that the electron is moving from O_23_ to C_27_, and the electron-hole coherence is stronger at T_1_ and T_2_. At 22 fs, the element converge at (10, 2), which means that the electron is back on A again, is the transition from N_10_ to N_2_. The electron transition at 40 fs is similar to that at 3 fs, focusing on the O_19_ and C_14_ of acceptor T_1_. The most clear is that at 50 fs, the elements have an obvious distribution on A, T_1_, and T_2_, illuminating that the charge is gradually transferred from A to T_1_ and T_2_ at this moment. The electron–hole coherence is present throughout the molecule. At 70 fs, the electron is concentrated on (19, 24) again, and the charge is transferred periodically. At 90 fs, the charge is concentrated in the T_1_ and T_2_ regions. At 100 fs, the element is again concentrated at (19, 24), ready to start a new round of periodic oscillation. During a period of 100 fs, it seems as though the electron is being transferred back and forth between the donor and the acceptor, and the electron–hole coherence varies periodically.

### 2.5. Hole–Electron Analysis and Marcus–Levich–Jortner Theory

**Figure 5:**
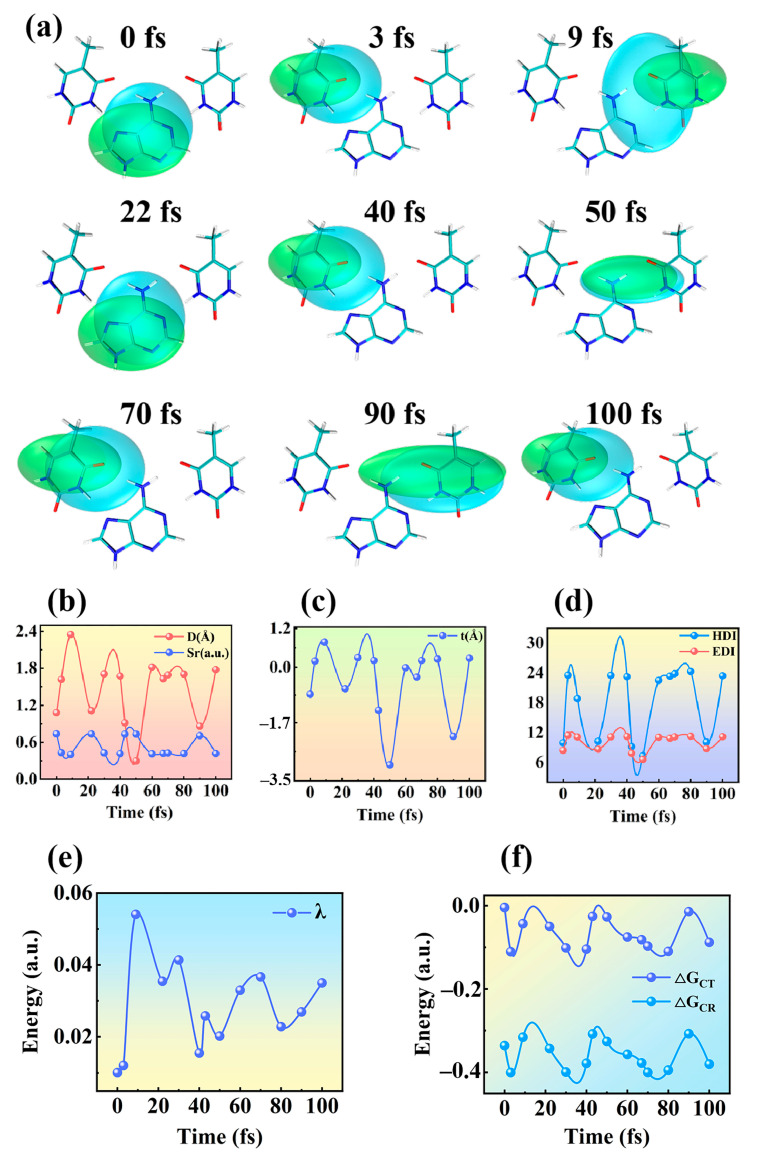
Dynamic evolution of hole–electron interaction analysis and Marcus theory of TAT base triplet with time. (**a**) The distribution of electrons (green) and holes (blue) over time. (**b**) The numerical variation of the electron–hole distance D and the corresponding Sr. (**c**) Dynamic evolution of numerical t of electron–hole separation degree. (**d**) The corresponding evolution of HDI and EDI. (**e**) Change in reorganization energy over time. (**f**) Evolution of free energy $\Delta G_{CT}$ and $\Delta G_{CR}$ over time.

Hole–electron analysis can investigate the transition characteristics of coherent charge transfer at different times, as shown in Figure 5a. The electrons are in green, and blue is used for holes. The corresponding parameters describing the electronic excited states are listed in Figure 5b–d. Where the D index is the hole–electron distance, the overlap of the hole–electron is represented by the symbol Sr and the t index indicates the degree of hole–electron separation. The findings demonstrate that the D index approaches the Sr index at 0 fs, 22 fs, 50 fs, and 90 fs, while the t index is unmistakably negative. Moreover, both the hole delocalization index (HDI) and electron delocalization index (EDI) are much smaller. This explains why their hole distribution and electron distribution are more uniform and have obvious delocalization characteristics. All this evidence proves that TAT is in a locally excited state at these times. In this kind of excitation, the main distribution range of hole–electron is approach, the overlap degree is very high, and the hole–electron distribution is not obviously separated. On the contrary, at 3 fs, 9 fs, 40 fs, 70 fs, and 100 fs, the difference between D is large. The electron–hole center distance is far away, which expresses that TAT is undergoing charge transfer excitation at these moments. The relatively small Sr index indicates that the hole–electron overlap degree is small and the hole and electron are highly separated. The t index is positive, indicating that hole–electron separation is obvious at this time. A high separation degree can effectively strengthen electron excitation and promote the occurrence of coherent electron transfer in TAT. The corresponding HDI values and EDI values are relatively large, and the degree of delocalization of the holes and electrons is low. This implies that these moments include charge transfer excitation. It is clear that coherent electron transfer depends, critically, on the local excited states and charge transfer states of TAT, which change frequently throughout time.

Marcus–Levich–Jortner expression is widely used to explain the dynamics of vibrational coupling and charge recombination in donor–acceptor systems [45,46]. The following is how the Marcus–Levich–Jortner expression functions [47,48,49]:

(2) $$K_{ET}=\frac{4\pi^{2}}{h}{\left(H_{DA}\right)}^{2}\frac{1}{\left(4\pi \lambda k_{B}T\right)}\sum_{\nu }\exp(-S_{\mathrm{eff}})\frac{S_{eff}{}^{\nu }}{\nu !}\exp(-\frac{{\left(\lambda +\Delta G+\nu \hbar \omega \right)}^{2}}{4\lambda k_{B}T})$$

where $K_{ET}$ represents the rate of electron transfer, $k_{B}$ reflects the Boltzmann constant, and *h* is the Planck constant. The reaction temperature and electron coupling strength of the system are expressed by T and $H_{DA}$, respectively. The reorganization energy and free energy of the system can be evinced in terms of λ and ΔG. Theoretically, it should be possible to account for all molecular vibrational frequencies $\omega_{eff}$, hence a list of all possible vibrational energy levels ν is required. Since the most efficient location for electron transfer is the donor and acceptor interface, the simplest method is to select the appropriate frequency and define the effective Huang–Rhys factor $S^{eff}$ as $S_{eff}=\sum_{i}S_{i}$ and $\omega_{eff}=\frac{\sum_{i}\lambda_{i}}{\sum_{i}S_{i}}$. The key physical quantities of electron transfer rate can be calculated based on the Marcus–Levich–Jortner theory. When the electronic state of the system changes, the internal reorganization energy changes because of the relaxation of the geometric structure. The intramolecular reorganization energy is represented by the following formula [50]:

(3) $$\lambda =(E_{0}^{-}-E_{-})+(E_{-}^{0}-E_{0})$$

Among them, $E_{0}^{-}$ and $E_{-}$ are the optimized negative electron energies based on the neutral molecular structure and the anionic structure, respectively. The energy of the $E_{-}^{0}$ and $E_{0}$ neutral molecular are optimized based on the anionic and neutral molecular structures. The reorganization energy is shown in Figure 5e at various times. The value of the reorganization energy *λ* oscillates regularly from 0.01 a.u. to 0.05 a.u., which further reveals the coherent property of the electron transfer process of TAT. The conformation of TAT in the excited state will change, which will cause the change in the free energy of the system. ΔG is the change in total free energy after the electron is transferred from the donor to the acceptor. ΔG can be described in two parts: one is exciton dissociation energy ($\Delta G_{CT}$) and the other is charge recombination energy ($\Delta G_{CR}$). The Rehm–Weller equation can be used to express the value of $\Delta G_{CT}$ [51]:

(4) $$\Delta G_{CT}=-\Delta G_{CR}-\Delta E_{0-0}-\Delta E_{b}$$

$\Delta E_{0-0}$ is the lowest excited state energy of the donor, and $\Delta E_{b}$ is the exciton binding energy. The function expression of $\Delta G_{CR}$ is as follows [52]:

(5) $$\Delta G_{CR}=E_{IP}(D)-E_{EA}(A)$$

$E_{IP}(D)$ represents the ionization potential of the donor, and $E_{EA}(A)$ reflects the electron affinity of the recipient. The calculation results are displayed in Figure 5f. Both $\Delta G_{CT}$ and $\Delta G_{CR}$ oscillate regularly over time, with surprisingly consistent trends. This shows that coherent electron transfer in TAT is driven by the strong nuclear–electron coupling.

## 3. Conclusions

In summary, we use ab initio molecular dynamics and Ehrenfest dynamics to simulate the coherent electron transfer of TAT base triplets. Interestingly, it is discovered that the TAT base triplet electron transfer exhibits clear periodic oscillation with a period of 10 fs. The Kohn–Sham eigenvalues, hydrogen bond strength, and transition density matrices with time also exhibit periodic oscillations of 10 fs. An RDG analysis shows that the peak position of the hydrogen bond fluctuates regularly between −0.040 and −0.056. A CDD analysis elucidates a gradual shift in charge distribution from the donor (A) to the acceptor (T_1_ and T_2_), and quantifies the maximum charge transfer proportion of 59.817%. TDM tracks and visualizes the specific distribution of the electron and hole. By examining the hole–electron separation and overlap, as well as categorizing the locally excited and charge transfer states, it is found that charge transfer excitation promotes the electron coherent transfer of TAT. The results of free energy and reorganization energy indicate that the strong nuclear–electron coupling vibration drives the coherent transfer of electrons. It provides a real-time demonstration of the short-range coherent electron transfer of DNA base triplets and offers a useful understanding of coherence effects in biological systems.

## 4. Computational Details

The microscopic process of coherent electron transfer of TAT is visualized using first principles [53]. The geometry of TAT base triplets was optimized using the Gaussian 09 package [54]. To obtain the optimized electronic structures, Becke’s three-parameter hybrid exchange functions were combined with the Lee–Yang–Parr (B3LYP) gradient-corrected correlation functional. The basis set was the triple-ζvalence (TZVP) with a single set of polarization functions [55]. Using the Octopus program, the optimized molecular configuration was simulated using Ehrenfest dynamics and the correlation real-time, time-dependent density functional theory (RT-TDDFT) equation was solved [56,57]. The specific calculation process is shown in Figure S4. We simulate the core electron using Troullier–Martins pseudopotential [58]. The nucleus was widely propagated in Ehrenfest formalism. The time-dependent Kohn–Sham equation is coupled to the nuclear motion equation using the Ehrenfest Hamiltonian. The Ehrenfest dynamics provide a quantum force term as the mean field in the classical equation of nuclear motion to explain the nuclear–electron interaction [59]. It is a good way to explain the ultrafast charge delocalization and rearrangement. In order to approximate the evolution operator, the approximate enforced time reversal symmetry algorithm is used, and the related Hamiltonian exponents are counted using Taylor series expansion [60]. The initial photoinduced electron configuration of Ehrenfest dynamics is produced by moving an electron from the highest occupied molecular orbital (HOMO) to the virtual lowest unoccupied molecular orbital (LUMO) (see Figure 1c and Figure S1). The linear response TDDFT verifies that these orbitals contribute the most to the dominant optical transition. The following formula is used to monitor the coherent charge transfer process over time [61]:

(6) $$PSD(r)=\rho^{\beta }(r)-\rho^{\alpha }(r)={\sum_{i}^{N/2}\left|\Phi_{i}^{\beta }(r)\right|}^{2}-{\sum_{i}^{N/2}\left|\Phi_{i}^{\alpha }(r)\right|}^{2}$$

Here, ρ represents the electron density, and α and β represent the type of spin. The number of electrons and the Kohn–Sham molecular orbitals are represented by N and $\Phi_{i}$, respectively. Although the total spin of the TAT base triplet is about 0, upon excitation, the spin distribution of the system will show α spin region and β spin region. By calculating the difference between α and β, we give the change in charge density with time to visualize these dynamics. From Tables S1 and S2, the total energy is converged to near 0.1 eV for a radius of 4.5 Å and a spacing of 0.15 Å, which produced a 122 Ry cut off. The step sizes of TAT are adjusted to 1 attosecond in order to compare the evolution of nucleus and electron throughout time correctly. The kinetic energy increases throughout the trajectory as a result of the relaxation from the Franck–Condon point, and the initial nuclear velocities are set to zero. In our simulations, the degrees of freedom for the electronic and nuclear systems propagate on the same time grid. Moreover, the analysis of the reduced density gradient, the transition density matrix, and the hole–electron coupling depends on the Multiwfn program and VMD software [62,63,64].

## Data Availability

Not applicable.

## Supplementary Materials

The following supporting information can be downloaded at: https://www.mdpi.com/article/10.3390/molecules28196802/s1, Different functionals and basis sets were tested and calculated, the infrared vibrational spectra changed over time, and the optimized TAT structure coordinates were displayed in the supporting information. Movie S1: The CDD of the simulated TAT from 0 fs to 100 fs. Table S1 Select different radius values corresponding to the energy. Table S2 Select different spacing values corresponding to the energy. Table S3 The maximum absorption peak of TAT was calculated by different functionals and basis sets (Exp = 260 nm [29,30]). Upper half: We change the functional, keeping the basis set TZVP. Lower half: We change the basis set keeping the functional B3LYP. Figure S1. The relative molecular orbitals and energy under different functionals. Figure S2. Evolution of infrared vibrational spectra of charge donor (A) and acceptor (T1 and T2) over time. Figure S3. The time-dependent dynamics of N-H bonds near the hydrogen bond grid is obtained with the Gaussian 09 package at the B3LYP (TZVP) level. The optimized coordinates of TAT.
