# Supplementary material for: Short-Range Charge Transfer in DNA Base Triplets: Real-Time Tracking of Coherent Fluctuation Electron Transfer
Source: Molecules. 2023 Sep 25;28(19):6802. doi: 10.3390/molecules28196802 (PMC10574627; doi:10.3390/molecules28196802)
Supplement: Supplementary file 1 [file molecules-28-06802-s001.zip › Supporting Information.pdf]

Supporting Information

Short-Range Charge Transfer in DNA Base Triplet: Real-Time Tracking of Coherent Fluctuation Electron

Lixia Zhu, Qi Li, Yongfeng Wan, Meilin Guo, Lu Yan, Hang Yin and Ying Shi*
Institute of Atomic and Molecular Physics, Jilin University, Changchun 130012, China

Email: shi_ying@jlu.edu.cn

Table of Contents

Table of Contents 2

Table S1 3

Table S2 4

Table S3 5

Table S4 6

Figure S1 7

Figure S2 8

Figure S3 9

Figure S4 10

The optimized coordinates of TAT 11

References 14

Author Contributions 15

Table S1 Select different radius values corresponding to the energy.

Radius (Å)	Energy (eV)
2.5	-6806.06873
3.0	-6808.0318
3.5	-6808.35663
4.0	-6808.41495
4.5	-6808.42451

Table S2 Select different spacing values corresponding to the energy.

Spacing (Å)	Energy (eV)
0.11	-6778.86763
0.12	-6779.09314
0.13	-6779.34191
0.14	-6779.23691
0.15	-6779.53078
0.16	-6779.63818

Table S3 The maximum absorption peak of TAT was calculated by different functionals and basis sets (Exp=260 nm[1-2]). Upper half: We change the functional, keeping the basis set TZVP. Lower half: We change the basis set keeping the functional B3LYP.

Functional	Absorption peak (nm)
B3LYP	254
CAM-B3LYP	235
PBEPBE	381
mPW1PW191	246
ωB97XD	234
Basis set	Absorption peak (nm)
TZVP	254
6-31G (d,p)	248
cc-pVTZ	253

Table S4 Specific proportion of charge transfer at different time.

	Charge transfer
0 fs	14.994 %
3 fs	3.169 %
9 fs	25.102 %
22 fs	10.796 %
40 fs	4.168 %
50 fs	59.817 %
70 fs	3.513 %
90 fs	27.363 %
100 fs	5.315 %

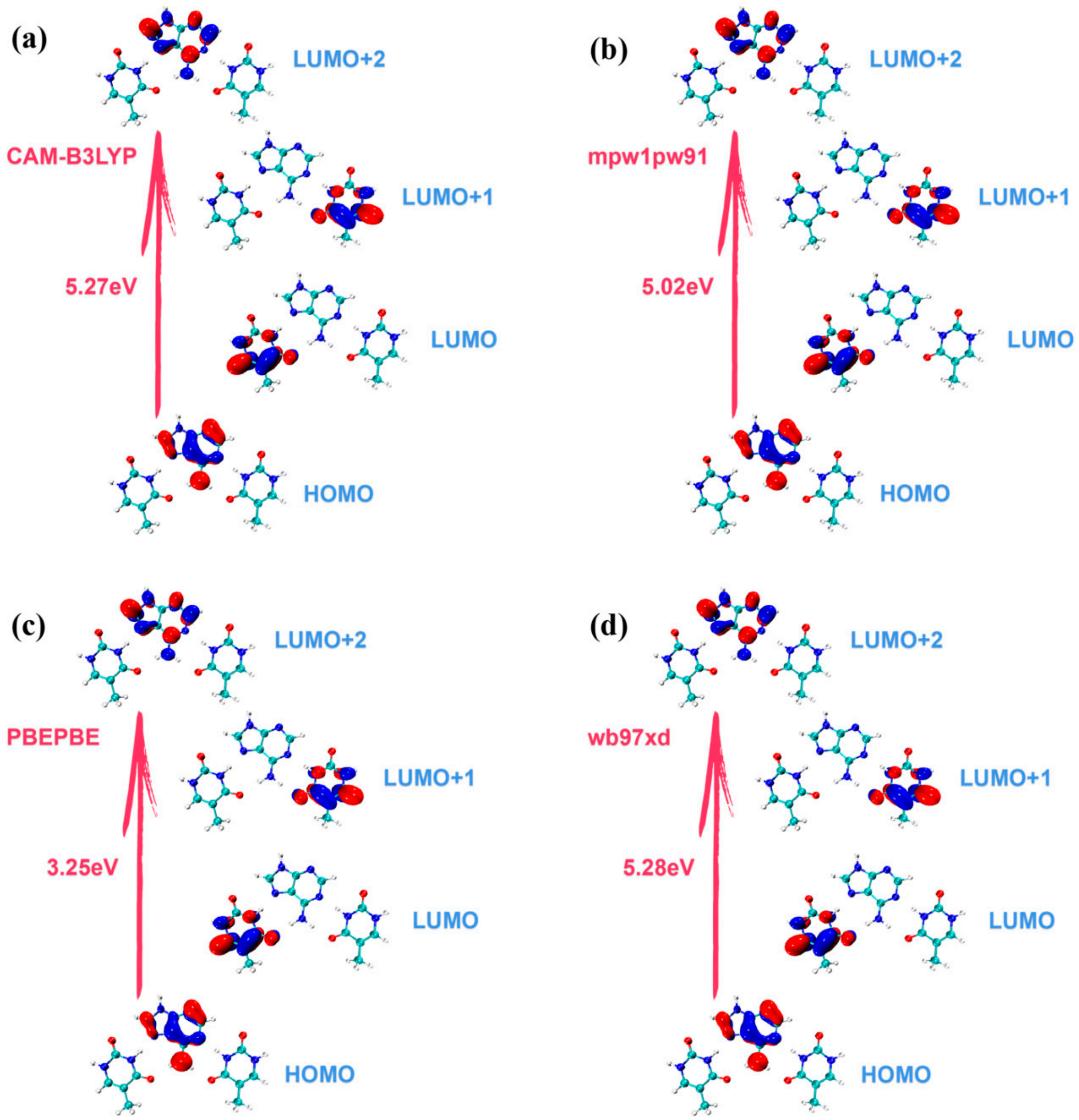

Figure S1. The relative molecular orbitals and energy under different functionals.

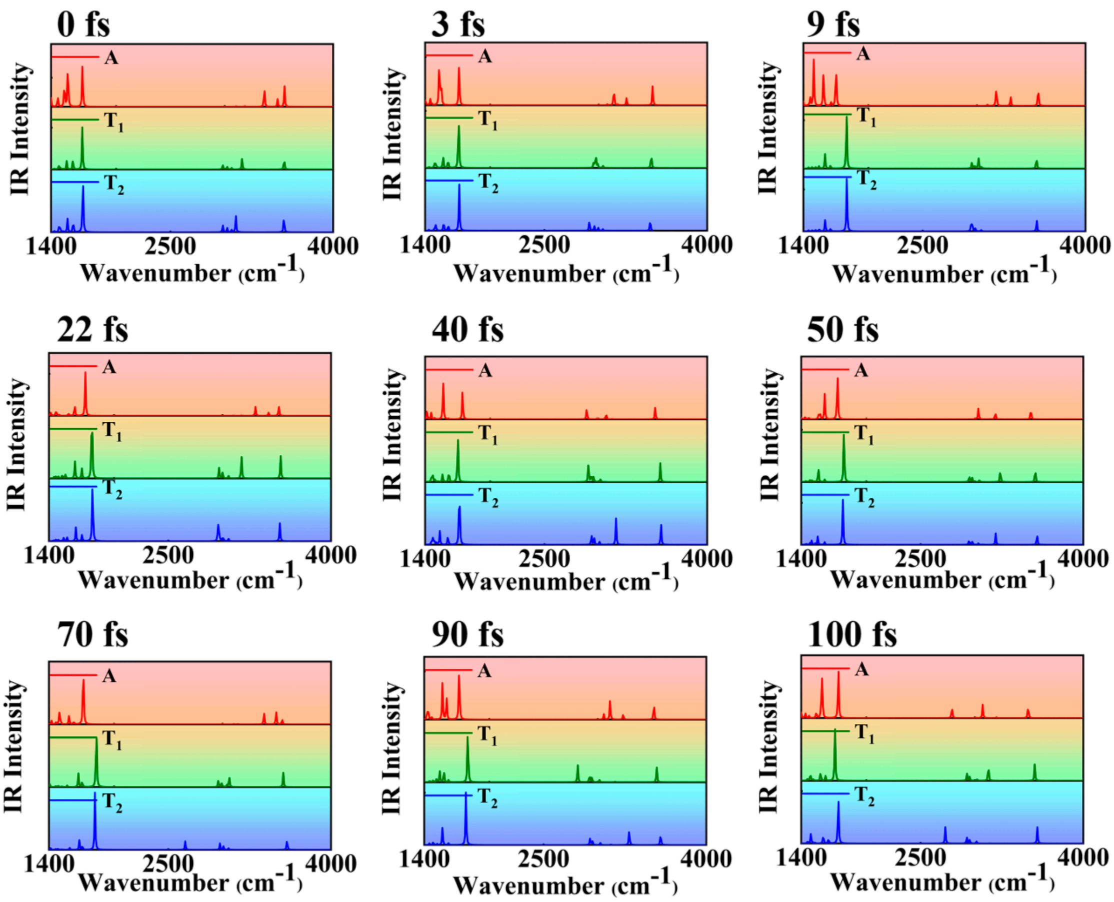

Figure S2. Evolution of infrared vibrational spectra of charge donor (A) and acceptor (T_1 and T_2) over time.

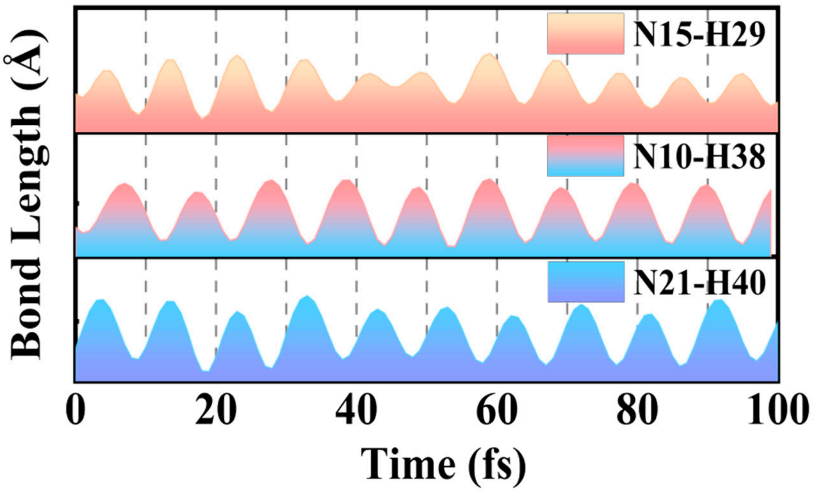

Figure S3. The time-dependent dynamics of N-H bonds near the hydrogen bond grid is obtained with the Gaussian 09 package at the B3LYP (TZVP) level.

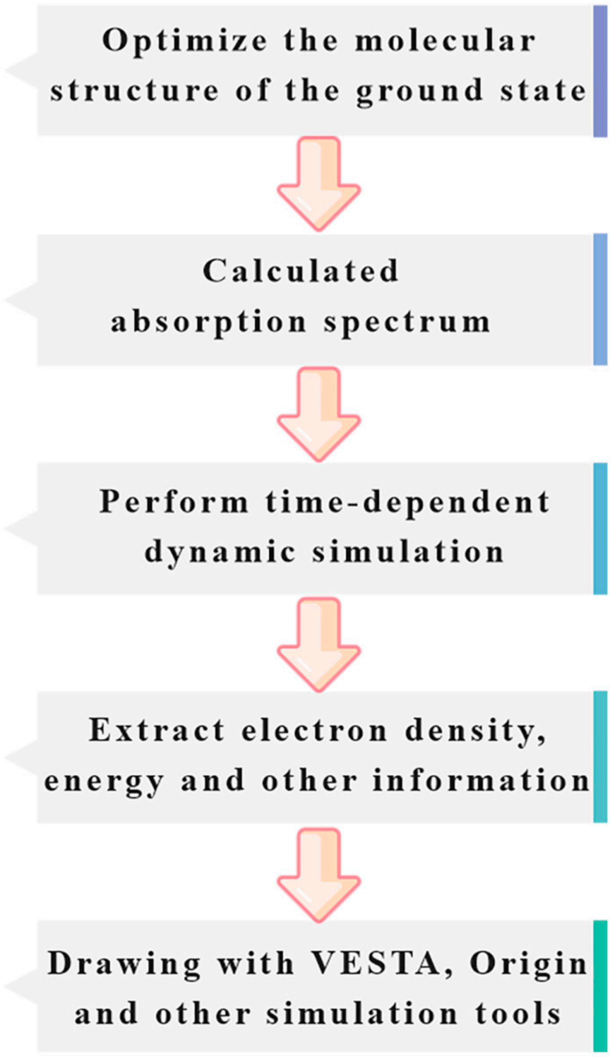

Figure S4. The calculation flow chart.

The optimized coordinates of TAT

C	-5.89783700	-2.17518100	-0.00087200
C	-4.62064200	-2.60547400	-0.00007800
C	-3.56198400	-1.59388000	0.00072600
N	-3.99034100	-0.27162900	0.00071700
C	-5.28873000	0.18239900	-0.00018800
N	-6.22855500	-0.83930400	-0.00095800
O	-5.60168200	1.35944300	-0.00029800
O	-2.36417200	-1.85814600	0.00143500
C	-4.22633100	-4.05023300	-0.00000900
H	-3.25076900	0.46115900	0.00092300
H	-6.73832000	-2.85715800	-0.00151600
H	-7.19201600	-0.54339900	-0.00146800
H	-3.61773800	-4.28745800	-0.87522400
H	-5.10478000	-4.69683000	-0.00069100
H	-3.61891400	-4.28770600	0.87595500
C	-0.67008600	1.95401300	0.00024400
C	0.38675900	1.01135200	-0.00029400
N	1.64417100	1.51654100	-0.00074500
C	1.84010000	2.84300300	-0.00061900
N	0.92363300	3.80549900	-0.00011900
C	-0.31262900	3.29981700	0.00026100

N	-2.04767100	1.82439700	0.00059000
C	-2.51046100	3.04902100	0.00083800
N	-1.50827800	3.98420600	0.00063500
N	0.21154200	-0.31053100	-0.00039500
H	2.87931500	3.15446200	-0.00098300
H	-3.55885300	3.30560200	0.00101900
H	-1.60954100	4.98655400	0.00095100
H	1.02677100	-0.92062400	-0.00071700
H	-0.71406500	-0.72933000	0.00010300
C	6.25019200	-1.63287200	0.00061100
C	5.06795500	-2.27982200	0.00026300
C	3.84864200	-1.46926800	-0.00043500
N	4.03432400	-0.09197100	-0.00063900
C	5.23517500	0.58234500	-0.00027100
N	6.34035200	-0.26105000	0.00037400
O	5.34130300	1.79351000	-0.00047000
O	2.71696400	-1.94543300	-0.00083200
C	4.93270900	-3.77137800	0.00052400
H	3.17191300	0.49783500	-0.00090000
H	7.19715600	-2.15726900	0.00113200
H	7.23617500	0.20076900	0.00055400
H	4.37554400	-4.11183800	0.87598000

H	5.91113800	-4.25369600	0.00113200
H	4.37644100	-4.11223200	-0.87535100

References

[1] A. Banyasz, T. Ketola, L. Martínez-Fernández, R. Improta, D. Markovitsi, *Faraday Discuss.*, **2018**, *207*, 181-197.

[2] F. D. Lewis, H. H. Zhu, P. Daublain, T. Fiebig, M. Raytchev, Q. Wang, V. Shafirovich, *J. Am. Chem. Soc.* **2006**, *128*, 791-800.

Author Contributions

L. X. Zhu and Y. Shi conceived the research idea. Q. Li, Y. Wan, Y. F. Wan conducted data analysis. L. X. Zhu wrote the original manuscript. M. L. Guo, L. Yan, H. Yin checked the manuscript. All authors discussed the results and commented on the manuscript.
